# Responding to COVID-19: Emerging Practices in Addiction Medicine in 17 Countries

**DOI:** 10.3389/fpsyt.2021.634309

**Published:** 2021-03-12

**Authors:** Florian Scheibein, M. J. Stowe, Sidharth Arya, Nirvana Morgan, Tomohiro Shirasaka, Paolo Grandinetti, Noha Ahmed Saad, Abhishek Ghosh, Ramyadarshni Vadivel, Woraphat Ratta-apha, Sagun Ballav Pant, Ramdas Ransing, Rodrigo Ramalho, Angelo Bruschi, Tanay Maiti, Anne Yee HA, Mirjana Delic, Shobhit Jain, Eric Peyron, Kristiana Siste, Joy Onoria, Saïd Boujraf, Lisa Dannatt, Arnt Schellekens, Tanya Calvey

**Affiliations:** ^1^School of Health Sciences, Waterford Institute of Technology, Waterford, Ireland; ^2^Department of Family Medicine, School of Medicine, Faculty of Health Sciences, University of Pretoria, Pretoria, South Africa; ^3^State Drug Dependence Treatment Centre, Institute of Mental Health, Pt Bhagwat Dayal Sharma University of Health Sciences, Rohtak, India; ^4^University of the Witwatersrand, Johannesburg, South Africa; ^5^Department of Psychiatry, Teine Keijinkai Medical Center, Sapporo, Japan; ^6^Addiction Services (SerD), Department of Territorial Services, ASL Teramo, Teramo, Italy; ^7^Department of Psychiatry, Ain Shams University, Cairo, Egypt; ^8^Drug Deaddiction and Treatment Centre, Postgraduate Institute of Medical Education and Research, Chandigarh, India; ^9^Waikato District Health Board, Waikato, New Zealand; ^10^Faculty of Medicine Siriraj Hospital, Mahidol University, Salaya, Thailand; ^11^Department of Psychiatry and Mental Health, Institute of Medicine, Tribhuvan University, Kathmandu, Nepal; ^12^Department of Psychiatry, BKL Walawalkar Rural Medical College, Ratnagiri, India; ^13^Department of Social and Community Health, School of Population Health, The University of Auckland, Auckland, New Zealand; ^14^Department of Mental Health, ASL Viterbo, Viterbo, Italy; ^15^Department of Psychiatry, All India Institute of Medical Sciences (AIIMS), New Delhi, India; ^16^Department of Psychological Medicine, University Malaya Centre of Addiction Sciences (UMCAS), Faculty of Medicine, University of Malaya, Kuala Lumpur, Malaysia; ^17^Center for Treatment of Drug Addiction, University Psychiatric Clinic Ljubljana, Ljubljana, Slovenia; ^18^Department of Psychiatry, Heritage Institute of Medical Sciences (HIMS), Varanasi, India; ^19^AddiPsy, Lyon, France; ^20^Department of Psychiatry, Faculty of Medicine Universitas Indonesia-Ciptomangunkusumo Hospital, Jakarta, Indonesia; ^21^Department of Psychiatry, College of Health Sciences, Makerere University, Kampala, Uganda; ^22^Faculty of Medicine and Pharmacy, Sidi Mohamed Ben Abdellah University of Fez, Fes, Morocco; ^23^Department of Psychiatry and Mental Health, Faculty of Health Sciences, University of Cape Town, Cape Town, South Africa; ^24^Radboud University Medical Centre, Nijmegen, Netherlands; ^25^Faculty of Health Sciences, University of the Witwatersrand, Johannesburg, South Africa

**Keywords:** COVID-19, drug policy, addiction medicine, substance use, behaviourial addictions, best practice, guidelines

## Introduction

Following the classification of the Coronavirus disease (COVID-19) as a pandemic by the World Health Organization (WHO), countries were encouraged to implement urgent and aggressive actions to change the course of the disease spread while also protecting the physical and mental health and well-being of all people. The challenges and solutions of providing prevention, treatment, and care for those affected with issues related to substance use and addictive behaviors are still being discussed by the global community. Several international documents have been developed for service providers and public health professionals working in the field of addiction medicine in the context of the pandemic (1–3), however, less is known about country-level responses. In the current paper we, as individual members of the Network of Early Career Professionals working in Addiction Medicine (NECPAM), discuss emerging country-level guidelines developed in the 6 months following the outbreak.

We identified a number of pertinent, country-level documents in the 17 countries represented here and we summarized country-level briefing notes, practice documents, guidelines, discussion papers and other documents containing recommendations on prevention, harm reduction, treatment, and care for people who use drugs (PWUD). Documents were identified in 12 out of the 17 countries. These documents are summarized and charted in Table 1. Additionally, several documents were under development at the time of our exercise in the Netherlands, Slovenia, and Paraguay and have not been included in this work. No specific documents or intentions to develop any were identified in Egypt, Uganda, or South Africa. Below we provide a summary of the identified documents.

**Table 1:** Country specific COVID-19 guidance documents for clinical practice in addiction medicine.

**Country**	**Author**	**Type**	**Topics**
India	AIIMS (All India institute of medical sciences, New Delhi	Guidelines	• TADs of buprenorphine and methadone (bi weekly or alternate days) • Take home doses to be managed by “responsible adults”
	Basu D, Ghosh A, Subodh BN et al.	SOP	• Hospital SOP for buprenorphine-naloxone TADs
	Indian Psychiatric Society	Position statement, guidelines	• Warns of potentially increased incidence of AOD withdrawal and associated complication • Advocates for seven-day TADs • Advocates for physical distancing in OAMT clinic • Discusses supply/travel restrictions and human resource issues
	Indian Psychiatric Society and National Institute of Mental Health and NeuroSciences	Guidance document	• Advocates for reducing admissions • Physical distancing guidelines • Tobacco use • Telemedicine for follow up • Discusses challenges associated with physical distancing in emergency case management
Indonesia	Ministry of Health		• Advocates for TADs • Increased use of telemedicine • Safety procedures including PPE
Ireland	Health Service Executive	Guidelines, guidance documents, SOPs	• Recommends expedited access to OAMT (using telemedicine where possible) • Increased TADs • Increased naloxone availability (all inducted patients to be offered prescription) • Changes in naloxone administration (preference for IM, chest compressions only unless specially trained and with special equipment) • Telemedicine for follow up • Details procedure for expedited emergency induction • Standard operating procedure for operating National Drug Treatment Center Pharmacy OAMT program • Outlines general procedures for operating NSPs • Supply management • Advocates for increased harm reduction • Discusses challenges associated with human resources • Recommendations for storage and handling of prescription medication • Recommendations for conducting addiction telemedicine consultations
Italy	Federazione Italiana Operatori Dipartimenti e Servizi Dipendenze (FeDerSerD)	Guidance documents	• Detailed hygiene practices • Reduction of services • Suspension of groups (unless physical distancing is possible) • Promotion of telehealth • TADs OAMT (1 month) • Reduction of urine testing • Care with breathalyzers • Guidelines for service delivery in prison • Increased availability of extended-release preparations
France	Ministères des Solidarités et de la Santé	Recommendations	• Advocates for easier access to OAMT and nicotine replacement therapies (NRTs) • Advocates for maintaining communication with patients using telemedicine and reserve in-person meetings for emergencies • Improved prescription renewal procedures
Japan	Ministry of Health, Labor, and Welfare.	Policy	• Procedures for expedited emergency induction • Increased TADs • Physical distancing
	Japanese Medical Society of Alcohol and Addiction Studies	Guidelines	• Warns of overuse of the internet, gambling, gaming and drinking at home
	The Japanese Society of Psychiatry and Neurology	Guideline	• Use of online-based self-help groups
Malaysia	Ministry of Health	SOPGuidance document	• Increased TADs • Mental health and psychosocial support in COVID-19: 1) For general population 2) For healthcare workers 3) For team leaders in health facilities 4) For care providers for children 5) For older adults, care providers, people with underlying health conditions • COVID-19 Management Guideline for special settings, including prisons, lockup and detection camps
	National Anti-Drugs Agency	Guidance document	• Use of online- and telephone - based counseling
Morocco	Moroccan Addictology Association	Guidelines	• Advocates for TADs, home deliveries and take home naloxone • Warns of particular interactions between methadone and hydroxychloroquine and chloroquine- QT interval • Vigilant stock management of methadone • General PPE and risk reduction/control measures
Nepal	Ministry of Health and Population and National Center of AIDS & STD Control	Guidelines	• Provisions for OAMT and other harm reduction services • Guidance for PWID harm reduction program including TADs for OAMT (upto 7 days), family involvement, social support unit and NSP. • Recommendation for continuing HIV services • PPE recommendations in ART centers and OAMT clinics • Guidelines for community-based care and community care center for PLHIV
New Zealand	Ministry of Health	Guidelines and Resources	• Advocates for linking of employment, addiction and mental health services • Advocates for addressing the housing needs of people with severe mental health and substance harm issues • Advocates for harm reduction approaches for substance use and gambling • Promotes education and raising awareness • Promotes mental health and addiction telemedicine support • Promotes access to self-help tools for substance use and gambling • Promotes inclusion of people with lived experiences of addiction in service design • Advocates for Maori specialist services and increased primary care services
	The Royal Australian and New Zealand College of Psychiatrists	Guideline and resources	• Advocates for increased TADs and reduced supervised dosing • Provides risk assessment and mitigation procedures for TADs • Outlines medication management procedures for ‘isolated' patients • Advocates for buprenorphine depot • Advocates for telehealth
Thailand	Department of Medical Services, Ministry of Public Health	Guidance document	• Practical guide for admission rehabilitation and follow-up • Individual treatment only; limit group treatment • Decreased admissions except for emergency (e.g. delirium tremens) • Use of telemedicine • Use of public health volunteers
	Department of Medical Services, Ministry of Health	Guidance document	• Limit admissions to severe emergency cases and advocate for screening for COVID-19 • Provide information regarding COVID-19 to patients • Limit family visit and group activity • Physical distancing in treatment centers
	Royal College of Psychiatrists of Thailand	Guidance document	• Recommendations concerning alcohol withdrawal for physicians, nurses, and public health personnel

Documents developed in Indonesia (4), Italy (5), and Nepal (6) discuss the use of personal and protective equipment (PPE). Malaysian (7), Moroccan (8), New Zealand (9–11), and Australian (12) organizations published documents which outlined risk assessment and mitigation practices. Documents in India (13), Malaysia (7), and Thailand (14, 15) discussed reducing admission of patients. Documents in India (16), Indonesia (17), and Japan (18) outlined strategies for maintaining physical distance in clinics and Standard Operating Procedures (SOP) were developed for isolation units in Ireland (19).

Italian (20) and Thai (15) documents discussed reducing addiction services and limiting group meetings. Documents in France (21), India (13), Italy (20), Ireland (19), Japan (22), Malaysia (7), New Zealand (11), and Thailand (15) advocated for the increased use of telemedicine to address the reduction in services.

Documents published in India (23) and Thailand (24) addressed substance withdrawal. The Thai document included strategies for the management of alcohol withdrawal that may have occurred due to local restrictions on alcohol sales. In Japan (22), there were discussions regarding the potential increase in the use of the internet, gambling, gaming, and higher prevalence of drinking at home during the COVID-19 pandemic.

Documents in France (21), Japan (25), and Ireland (26) described emerging practices of expedited access to opioid agonist maintenance treatment (OAMT). Documents in Ireland (26), India (23), Italy (20), Japan (25), Malaysia (7), Morocco (8), Nepal (6), and New Zealand (11) advocated for increased take-home doses (TADs) of OAMT. SOPs for buprenorphine-naloxone TADs in a hospital context have been developed in India (27) and documents in Indonesia (17), Nepal (6), Malaysia (7), and Italy (5) advocated for increased TADs of OAMT to 7 days, 14 days and 1 month, respectively. An Irish document (26) advocated for prescriptions for naloxone for all new OAMT patients and changes in the naloxone administration procedure (move toward intramuscular injection and chest compression in the absence of specialized equipment during opioid overdose interventions).

Guidelines, SOPs and recommendations in Nepal (6), Ireland (28, 29), and France (21), respectively, have also advocated for increased access to harm reduction services. In New Zealand, guidelines addressed practices of adopting a health equity/social determinant lens, developing culturally and trauma informed approaches, awareness, and education efforts, development of self-help resources and the inclusion of people with lived experience of substance use and gambling into the evaluation of interventions (10, 11).

## Discussion

A range of practices have been suggested at the country-level to deal with the challenges brought about by the ongoing pandemic. These include those around mitigating the spread of the corona virus, managing the risks associated with lockdown policies and changing trends in substance use and addictive behaviors.

In order to limit the spread of COVID-19, guidance has been drawn up to limit in-person meetings, physical support meetings, and contact time with physicians. Guidance suggests that this be operationalised through shifting services online, increased availability of TADs of OAMT, increased duration of TADs and increased availability of naloxone and injecting equipment allocations. Protocols have also been drawn up for the operation of clinics and outreach services for patients in isolation.

Several potential negative effects associated with the pandemic and resulting lockdown procedures have been identified which may require service adaptions. These include increased risks of substance withdrawal (30), access to service issues and potential changes in trends related to gambling, gaming, and internet related disorders. Several guidance documents discuss meeting these challenges through increased access to TADs, expedited access to OAMT and increased availability of online-based self-help groups and other services (11, 17–30). The increased commitment to TADs, telemedicine and access to harm reduction supplies are likely to address several issues brought about by the pandemic for people who use opioids and/or inject drugs. However, few documents explicitly discuss the increased availability of harm reduction supplies (for example, naloxone and injecting equipment) and service adaptions for people who use non-opioid drugs and/or engage in addictive behaviors (such as gambling and gaming) continue to be neglected by most documents.

There are also concerns regarding the implementation of COVID-19-related policy documents as a recent global survey indicates that among 130 countries, 60% reported disruptions to mental health services for vulnerable people, 67% reported disruptions to counseling and psychotherapy, 35% reported disruptions to emergency interventions, and 30% reported disruptions to access for medications for mental, neurological, and substance use disorders (31). The combination of a reduction in the availability of services, increased reliance on telemedicine, physical distancing protocols, and travel restrictions may exasperate underlying health inequities in terms of access to addiction services (31–34). This seems to disproportionately affect the most marginalized and socioeconomically disadvantaged patients (32) who may lack access to internet-enabled devices, sufficient internet, the necessary private spaces to engage in telemedicine and means of transport to services.

The lack of representation of country-level documents from the Americas, Eastern Europe, the Middle East, Africa, and other regions is a limitation of this paper. Future research should document emerging practices in additional regions and monitor and evaluate the implementation of country-level policies. Country-level documents may be useful as they may allow clinicians to adapt to their given local context. Such documents should consider best emerging practices as it relates to issues surrounding a wide range of substances, addictive behaviors, harm reduction, and health inequities exasperated by the pandemic and restrictions.

## Author Contributions

FS and TC developed the initial draft of the document. The commentary was then reviewed by MS and NM. All authors subsequently reviewed their sections and the overall document. All authors identified their own local documents or confirmed the lack of their existence.

## Conflict of Interest

The authors declare that the research was conducted in the absence of any commercial or financial relationships that could be construed as a potential conflict of interest.
